# Acknowledgment to Reviewers of *Medicina* in 2021

**DOI:** 10.3390/medicina58020258

**Published:** 2022-02-09

**Authors:** Medicina Editorial Office

**Affiliations:** MDPI AG, St. Alban-Anlage 66, 4052 Basel, Switzerland

Rigorous peer-reviews are the basis of high-quality academic publishing. Thanks to the great efforts of our reviewers, *Medicina* was able to maintain its standards for the high quality of its published papers. Thanks to the contribution of our reviewers, in 2021, the median time to first decision was 22 days and the median time to publication was 39 days. The editors would like to extend their gratitude and recognition to the following reviewers for their precious time and dedication, regardless of whether the papers they reviewed were finally published:
Aalap ChokshiJoan Carles Escolà-GilAbdelsamed ElshamyJoana DamásioAbdolhassan KazemiJoanna BogusławskaAbedalrhman AlkhateebJoanna Dolar-SzczasnyAbhilash VenugopalanJoanna KonopińskaAbhiram KannegantiJoanna PawłowskaAbhishek Kumar SinghJoanna PocztaAbhishek SethJoanna Rupa-MatysekAboozar MonavarfeshaniJoanna SłomkoAbraham PouliakisJoanna StefanowiczAchim FranzenJoanne FieldingAda AitaJoão M. SantosAdam DaragóJoão MesquitaAdam JanasJoão Pinto-De-SousaAdam ReichJoao RequichaAdam WisniewskiJochen GilleAddisson SalazarJohann ZwirnerAdel El BoueizJohanna T. KurzhagenAdel HelmyJohannes FleckensteinAdelaida AvinoJohannes J. DuvekotAdeyinka AdebayoJohannes LinxweilerAdil AsgharJohannes SchalamonAdina BrahaJohn EllulAditi KulkarniJohn F. BlaikleyAdolfo Di FioreJohn GriniatsosAdrian C. BalicaJohn K. YueAdrián CurtoJohn P. BowmanAdrian SchultzJohn Sukumar AluruAdriano FriganovicJolanta E. LosterAdrija HajraJolanta JaworekAgata M. NiewczasJolanta Pytko-PolończykAgata ZmijewskaJolanta SiudikienėAgata ZnamirowskaJon B. SuzukiAgnieszka GachJonathan D. KarpAgnieszka Gasowska-BodnarJonathan FusiAgnieszka JankowskaJonathan M. SorgerAgnieszka Madej-PilarczykJonathan RobinsonAgnieszka Różdżyńska-ŚwiątkowskaJonathan RothAgnieszka Szlagatys-SidorkiewiczJongkil JooAgnieszka WareńczakJoohyung LeeAgostino GaudioJoon Hyuk ChoiAgustín Díaz-ÁlvarezJoonhong ParkAhmad MirzaJooyoung KimAhmed AbdelghanyJorge DazaAhmed BakryJorge Moreno CharcoAhmed KhaledJosé A. CochoAhmed MohsenJosé Antonio AlarcónAhmet Yığıt ÇakıroğluJosé Antonio SanchesAida PetcaJosé Avendaño-OrtizAikaterini AmanitiJosé Bernardo Noronha MatosAiko NagaiJosé Carlos Pachon MateosAinoa Roldán AliagaJose CarugnoAishwarya GurumurthyJosé De Jesús Rangel MagdalenoAjay PalJose Di DiegoAkanksha SinghJosé Francisco López-GilAkhilanand ChaurasiaJosé Francisco Rodríguez-VázquezAkira MiyauchiJosé H. TeixeiraAkira UmemuraJose J. Gil-CosanoAkram MohammedJose Jesus Castro-SchezAkshata NaikJose L. Gómez-UrquizaAkshay KumarJose Luis Gandia FrancoAlak MannaJose Luis Gonzalez-MontesinosAlan G. FinkelJosé Luis Lázaro-MartínezAlarico ArianiJosé Luis MansurAlba Cecilia Ruiz-GaitánJosé Luis PuglisiAlban Fouasson-ChaillouxJosé M. Palacios-JaraquemadaAlbert WabneggerJose M. PrietoAlberto BalduzziJosé Manuel Almerich-SillaAlberto BenussiJosé Manuel López GarciaAlberto BollettaJosé Maria LassoAlberto Di NapoliJosé María Remes-TrocheAlberto FerrareseJose N. Sancho-ChustAlberto RighiJose PreciosoAlberto Rodríguez-ArchillaJose PuzoAldona KowalskaJose Sanchez-AlcazarAlejandro Herreros-PomaresJosep GallifaAlejandro Martínez-ÁguilaJosep M. AlegretAlejandro Martínez-RodríguezJoseph M. NorrisAlejandro Sanz-ParisJoseph PaydarfarAlejandro TelloJoseph VadakaraAleksandar DiklicJosh SchroederAleksandra FucicJoshua RempellAleksandra GaseckaJosip A. BorovacAleksandra JoticJosipa VlainićAleksandra KristoJosko BozicAleksandra SzylińskaJoy A. GreerAlen OstojićJozef SimenkoAleš TomažičJuan Antonio MorianaAlessandra AlciatiJuan Bautista De SanctisAlessandra BorsettiJuan I. Pérez-CalvoAlessandra CostanzaJuan J. CerrolazaAlessandra M. FerraroJuan Manuel Marquez-RomeroAlessandra Verzelloni SefJuan Manuel PericasAlessandro AntonelliJuan P. RodrigoAlessandro ArenaJuan Pedro Martínez RamónAlessandro CastiglioneJuan Rodríguez MansillaAlessandro De SireJuha TaavelaAlessandro FeolaJulia Guerrero-GironésAlessandro GambellaJulián Gustavo LleraAlessandro GranitoJulie HuntAlessandro MalobertiJulien FrandonAlessandro MeduriJulien HarocheAlessandro NotaJulio CostaAlessandro RizzoJun FukiharaAlessandro ScoppolaJun NishikawaAlessandro VittoriJun WatanabeAlessia TognettoJunfeng WangAlessio GambinoJung-Joon ChaAlex M. HankeyJunichi OmuraAlex MichelsJūratė BalčiūnienėAlex R. ChangJuste KaciulyteAlexa HollingerJustyna KowalskaAlexander ChanJustyna Mikula-PietrasikAlexander Dimitrov ShinkovJwu-Lai YehAlexander ShpakJy-Ming ChiangAlexandr CeasovschihJyothsna ManikkathAlexandra CaziucJyrki VuolaAlexandra FrogoudakiK. S. BenschopAlexandra Maria Giovanna BrunassoKaining ZhiAlexandre Docampo‐SimónKajetan GrodeckiAlexandre JoostenKajo BućanAlexandros RovasKalathil K. SureshkumarAlexandru Andrei MunteanKalyan SaginalaAlexandru BurlacuKamal Kant SahuAlexandru PopaKamil GillAlexandru UliciKamil M. FramAlexei N. SuminKari Bjerke Batt-RawdenAlexey SarapultsevKarin S. WildiAlfio FerlitoKarl BechterAlfonso Del CuvilloKarl-Heinz WidmerAlfonso Martínez-NovaKarol KamińskiAlfred SimonKarol KarneckiAlfredo VellidoKarol RycerzAli BoolaniKarolina GerrethAli Ekrem YesilkanalKarolina Kędzierska-KapuzaAli MarieKarolina KossakowskaAli MobasheriKarolina ŁagowskaAli NadzalanKarri SilventoinenAli S.M. JawadKarsten RuscherAlice CasariKarthik RagunathanAlice Lay Hong ChuahKassapa EllepolaAlice VerticchioKatarzyna DomaszewskaAlicia Martínez-VareaKatarzyna FabjańskaAlicja FormaKatarzyna GrzelaAlicja KluczykKatarzyna HojanAlina VedutaKatarzyna Jezierska-WozniakAlinoë LavillaureixKatarzyna KaczyńskaAljaz HojskiKatarzyna KusnierzAllister GibbonsKatarzyna ŁubiechAllyson CochranKatarzyna MichalekAlok KumarKatarzyna NeubauerAlqahtani Bader AliKatarzyna PawlakAltaf MohammedKatarzyna PiotrowskaAlvaro Lopez-SamanesKate M. ChittyAmaju A. IkomiKatharina RumpAmanda V. SardeliKatherine J.E. SattlerAmandine JullienneKatherine SemrauAmbarish P. BhatKatrina HutchinsonAmbra BuschiazzoKazimierz KarolewskiAmbrogio P. LonderoKazuhide OhtaAmelia BrunaniKazuhiko HoriguchiAmelia Maria GamanKazuhiro P. IzawaAmelia PietropaoloKazuhito SuzukiAmer HarkyKazuki ShimizuAmirah GozaliKazumichi FujiokaAmit LadaniKazushige NireiAmit Satish BhattuKazuya InokiAmos LalKedar GhimireAmy L. ShaverKeechilat PavithranAna BorovečkiKehinde ObamiroAna DascaluKei SaitoAna GorostidiKei YamamotoAna Luísa De Sousa-CoelhoKelvin Ian AfrashtehfarAna Marchena RodríguezKen KatoAna S. PejčićKenichiro HiraokaAna Savic-RadojevicKenji HayashidaAna TomasKenji KanenishiAna Vujaklija BrajkovićKenneth A. AndreoniAnamaria BechirKensuke MitsunariAnand Prakash SinghKenta FurutaniAnandharajan RathinasabapathyKentaro OhkoAnastacia KudinovaKentaro YoshidaAnastasia KotanidouKentaro YoshiokaAnastasia ThanopoulouKeunBaDa SonAnca Marina CiobanuKe-Vin ChangAnca SimionescuKevin KempAndaleb KholmukhamedovKevin LouisAnders Christian LarsenKevin MeestersAndras BikovKevin RolfeAndraz DovnikKeyur VoraAndre CastroKhalid Sossey-AlaouiAndre HussKi Ho SeolAndré NovoKi Wha ChungAndre RodackiKiani KiandokhtAndre WirriesKi-bae HongAndrea AlexandreKiichi HirotaAndrea AnderloniKin M. YuenAndrea Ascoli MarchettiKiran PanickarAndrea BaragettiKirk PappanAndrea CarsettiKissy Guevara-HoyerAndrea CarugnoKitamura MineakiAndrea CortegianiKivanc GorguluAndrea De VitoKiyoshi MoriAndrea Del FattoreKlara SaczukAndrea Ghelli Di RoràKlaus Edgar RothAndrea LisottiKlaus GroschnerAndrea ManciniKlemens BuddeAndrea MingoliKo OkumuraAndrea PantaloneKoh KitagawaAndrea PiccinKohki OkadaAndrea R. PolariKoichiro MoriAndrea RiboliKoji OtaniAndrea RoccuzzoKonosuke NakajiAndrea SambriKonstantin Johannes ScholzAndrea SantarelliKonstantinos ApostolouAndrea SistiKonstantinos DimasAndrea StoccoroKonstantinos DitsiosAndreadi AikateriniKonstantinos KatogiannisAndreas ErfurthKonstantinos NatsisAndreas GatherKonstantinos SidiropoulosAndreas KochKoren E. RoachAndreas LipphausKrešimir GalešićAndreea AvasiloaieiKrešimir RotimAndreea-iren SerbanKripa ShankarAndrei LucaKrishna Kishore UmapathiAndrej ThurzoKristie SpencerAndrés CombaliáKristina DaunoravicieneAndrew BatemanKristine M. KrajnakAndrew JinKrzysztof KrystaAndrew LadwiniecKrzysztof W. ReczuchAndrew NorrisKuljit SinghAndriy MarusykKunal DhimanAndrzej L. ChamieniaKunihisa MiwaAndrzej P. OkoKun-Tsung LeeAndrzej PiotrowskiKylie GwynneAndrzej PolańczykKyongsong KimAndy PickettKyoung Soo KimAndy Wai Kan YeungKyoungmin ParkAneta OlszewskaKyung Eun LeeAnette KjeldsenKyung-mi KimAngel Romero ColladoLadislav ŠtěpánekÁngel Vicario-MerinoLars NorgrenAngela CartaLars P. KamolzAngela RosenbohmLars SmåbrekkeAngelika TerbuchLars-Peter KamolzAngelo ArmandiLászló SzabóAngelo AscaniLaura CominiAngelo SabagLaura FroehlichAngelo TroedhanLaura Fusar-PoliAngelos Arfaras-MelainisLaura HottenrottAngelos SikalidisLaura InfantiAniello MaieseLaura MangiaviniAnil K. VermaLaura MontejoAnil Ufuk BatmazLaura Sánchez-MuñozAnil VermaLaura-María Compañ GabucioAnish MukherjeeLauren B. NosanovAnja Haase-FielitzLaurent MetzingerAnju KumariLaurentiu PirteaAnn M. BergerLawrence JudgeAnn Sophie BohneLeah GramlichAnna BizońLee Ann Laurent-ApplegateAnna BrzeckaLee Gillian Hiu ManAnna CliffeLee Seng KhooAnna DubaniewiczLeena KadamAnna Janas-NazeLeigh A. KotzéAnna JanowiczLeja Dolenc-GroseljAnna KnightLéo RazakamanantsoaAnna LipertLeonardo OggianoAnna ManiaLeonardo RestaAnna Nele HerdinaLeonidas IoannouAnna NordenströmLewis CarpenterAnna Olasińska-WiśniewskaLeyla Safta-ZecheriaAnna Paradowska-StolarzLiana PlesAnna RaciborskaLidia GaffkeAnna RzepakowskaLiliana SerwecinskaAnna S. TzortziLiliana Szyszka-SommerfeldAnna StaniszewskaLily ChuAnna StarzyńskaLin ChengAnna SynakiewiczLina YoussefAnnafranca CavaliereLinda ChungAnnalisa CarlucciLinda TizekAnnalisa ChiavaroliLindsay S. SchenkelAnnalisa GuaragnaLingxiao XieAnnarosa Del MistroLionel ArrivéAnnarosa FloreaniLior ShapiraAnne FasslLirong WangAnnelie-Martina WeinbergLisa LungaroAnnette B. PfahlbergLivio VitielloAnthony DoyleLorenz LeitnerAnthony LemaireLorenzo GittoAnton GisteråLorenzo RicciAnton J. M. LoonenLorenzo RusticoAnton MoritzLorenzo ScappaticcioAntonella ArgentieroLorenzo TonettiAntonella GalloLouis KreitmannAntonietta FrancoLovorka BilajacAntonina SidotiLuboslav StárkaAntonino MazzoneLuca BruschiniAntonio BragaLuca Di GianfrancescoAntonio GattoLuca MagistrelliAntonio Jesús Martínez-OrtegaLuca RevelliAntónio M. G. BaptistaLuca Salvatore De SantoAntonio MafriciLuca ValerioAntonio Martinez-LopezLucia RomanoAntonio Palazón-BruLucia TattoliAntonio RomanelliLuciano BissolottiAntonio RussoLucio MangoAntónio Sérgio SilvaLuigi Angelo VairaAntonio Simone LaganàLuigi BarbatoAntonio VitielloLuigi BennardoAntonio ViúdezLuigi CappannoliAntoon LigtenbergLuigi CarboneAntti RannikkoLuigi FontanaAnubhav KanwarLuigi GianturcoAnusha PenumartiLuigi LoscoArgelia Medeiros-DomingoLuigi MeccarielloAri KoivistoLuigi PisaniAria NouriLuigi SantacroceArianna LaoretiLuigia De FalcoArif CanakogluLuis A. QuinonesAristeidis H. ZibisLuis Albendín-GarcíaAristeidis MyrkosLuís Carlos MatosArmond DaciLuis CordovaArnard Van Der ZwanLuis Del ValleArne KienzleLuis Ignacio González-GranadoAroa Tardáguila-GarcíaLuis NaculArpad KarsaiLuis RodrigoArshi KhanamLuis Suso-MartíArtur PasternakLuisa BayArturo BonomettiLuis-Guillermo Oliveros-LópezArturo CasadoLuiz Flávio Cordeiro FernandesArturo CesaroLukas RasulićArturo LópezLukáš ŠkoloudíkArul ChandranLukas ZakArvin AraniŁukasz ŁapajArvind NegiŁukasz MałekAsbjørn ÅrøenŁukasz PaczesnyAsha KumariLuminiţa Maria NicaAshok MandalaLy NguyenAshvin R. JaiswalMaciej Adam ChęcińskiAsif KhaliqMaciej J. KotowskiAsim KichlooMaciej MichalikAsja ČelebićMafalda LaranjoAssia DjemaMagali BarbieriAthanasia PapazafiropoulouMagdalena BaymakovaAthanasios TragiannidisMagdalena DobrolinskaAthina EconomouMagdalena Kocot-KępskaAtsuhiko YagishitaMagdalena KostkiewiczAtsushi KohgaMagdalena Lebiedzinska-ArciszewskaAtsushi KohyamaMagdalena SobieskaAttila BursicsMagdalena StaszczakAttila FrigyMahmoud NaguibAttila RokaMainul HaqueAttilio CastaldoMaite E. Rivera GorrínAudrey ChanMaja ValićAudrey SirventMakda GebreAudrius DulskasMakoto EndoAurel Popa-WagnerMakoto OeAurélie LadangMalgorzata KolodziejAurélie Tchoghandjian-AuphanMałgorzata Kulesa-MrowieckaAurelien BustinMałgorzata NitaAušra MongirdienėMałgorzata SobieszczańskaAvinash AujayebMalgorzata Trofimiuk-MüldnerAydin Bordbar-KhiabaniMalissa A. MulkeyAyman MahmoudMamta ShahAynur Emine CicekcibasiManabu SakuraiAyokanmi OreMandeep Singh DhillonAyumi SugiuraManel Sabaté TenasBabett RamsauerManfred SchmidtBa-Bie TengManiselvan KuppusamyBahareh AzimiManish AdhikariBahil GhanimManja M. ZecBakri M. AssasManlio SantilliBalázs BenyóManuel Gómez-GuzmánBaptiste LamarthéeManuel Jiménez NavarroBaran A. ErsoyManuela Adelinde AschauerBarbara BrognaManuela FarrisBarbara LisowskaManyoo A. AgarwalBarbara Mara KlinkhammerMaosen WangBarbara MognettiMara P. SteinkampBarbara Ruszkowska-CiastekMarc DorenkampBarbara SpanòMarc HilhorstBart HattinkMarc Nicolai BuscheBartłomiej KuleszaMarcel HanischBartosz DalewskiMarceli ŁukaszewskiBartosz GoneraMarcello CandelliBartosz MałkiewiczMarcello MocciaBartosz PulaMarcello RomanoBaruh PolisMarcelo Sousa SilvaBastian FischerMarcin CzechBeata Bujak-GizyckaMarcin JaneckiBeata Sarecka-HujarMarcin SamiecBeatrice BenattiMarcin WłodarczykBeatriz Gómez-MartínMarco AgusBeatriz MerinoMarco AnnunziataBehnam NabavizadehMarco AtteritanoBehnam RashidiehMarco BiffoniBen CostelloMarco CascellaBen GrayMarco ChiostriBeniamin Oskar GrabarekMarco DolciBenjamin E. Nilsson-PayantMarco FraccalvieriBerardino PorfírioMarco GuzzoBernhard BiersackMarco Maria PascaleBettina ZelgerMarco MontiBhupesh SinglaMarco PennazioBi O. JeongMarco PicardiBiagio MorettiMarco PonzettiBiagio RaponeMarco PossanziniBianca De Almeida PitittoMarco TallaricoBiljana BufanMarek FoksińskiBirgit BabitschMarek JoukalBirgit GartnerMarek PaulBishoy Y.A. El-AaragMarek SanakBlaise PierrehumbertMargit L.H. RiisBlanca XicoyMaría A. Pérez-HerreroBlanda Di LucciaMaria Addolorata BonifacioBlanka StiburkovaMaria ContaldoBlase BillackMaria DascaluBłażej CieślikMaria De Lourdes PereiraBogdan DorofteiMaria DudziakBogdan SoceaMaria Elena Vila-SuárezBojan ZarićMaria Elisabetta BaldassarreBoohwi HongMaria GazouliBorkowska AlinaMaria GiannìBortolo MartiniMaria Grazia MancinoBoyko GueorguievMaria IbrahimBradley P. AnderMaría Iniesta CuerdaBranislava A. MilenkovićMaria Irene BelliniBreffini C. AnglimMaría Jesús Alvarez CuberoBrendan CarvalhoMaría Llorián-SalvadorBrett G. JeffreyMaria Luigia RandiBrian OlshanskyMaria Morgan-BathkeBrienne A. McKenzieMaria MorrisseyBrigida IorioMaria SarapultsevaBrigita MiežienėMária ŠimaljakováBrigitte WilczekMaría T. AndrésBrijeshkumar PatelMaria-Cleofé ZaragozáBrîndușa CimpocaMariaenrica TinèBror Folke SjöbergMaria-Fernanda Lorenzo-GómezBruno BaršićMariama AkodadBruno Del SetteMariana Arias AvilaBruno FiondaMariana TudoranBruno MinottiMarianna LeopoulouBruno SalvatiMariano MascarenhasBryan E. DenhamMariano MastinuBulat RamazanovMariarita BrancaccioBumsoo AhnMarie Lue AntonyBungo OtsukiMarie Øbro FosbølByung-Kwan SeoMarija M. BožićCamelia DiaconuMarika MokouCameron HillMarika QuadriCanan KuscuMarika TardellaCarlo BizMarina LaborCarlo CavaliereMarina SartiniCarlo ContiniMario BilićCarlo Cosimo CampaMario F. ScaglioniCarlos David Gómez-CarmonaMárió GajdácsCarlos Fernando MourãoMario García-SuárezCarlos López-de-CelisMario GruppoCarlos Martin LlorenteMario LucianoCarlos Navarro CuéllarMario Mendoza-SagaonCarmen PanaitescuMarios PapadakisCarmine IzzoMarish OerlemansCarmine NovielloMarisol CueliCarol JohnstonMarius CostacheCarolina De CiuceisMariusz DruźbickiCarolina NegreiMariusz GoniewiczCaroline Ann AbbottMariusz NiemczykCaroline JadlowiecMariusz SikoraCarolyn Joy SachsMarjolaine BaudeCaspar Grond-GinsbachMark EidelmanCaterina DelceaMark Elisabeth Theodorus WillemsCaterina LiciniMark F. SommerfeldtCatherine Weikart YeckelMarko VelepicCecilia BoveMarkus KamlerCecilia PerinMarkus ReiserCees NoordamMarkus RuppCesare D’AmicoMarkus Tobias GrabbertCezar HonceriuMarkus ToelleChaeSeong LimMarshall S. RileyChandra Sekhar BathulaMarta Dyszkiewicz-KonwińskaChang Gue SonMarta GromichoChang-min LeeMarta Migocka-PatrzałekChangwon JeongMarta Nowakowska-KotasChan-Young KwonMarta OpalińskaCharalampos LoutradisMarta PelczyńskaCharles D. BurgerMarta SochockaCharles E. MillerMarta Wysocka-MincewiczCharles MarksMartha Alejandra Morales-SánchezCharlotte HoybyeMartha J. FalkensteinCharlotte Toftmann HansenMartín A. MaraschioCharu KothariMartin BakhtCheng JiangMartín Barra-LópezChenglei LiMartin Bjørn StausholmCheng-Yang ChouMartin KoestenbergerChen-Hua LiuMartin ScharffenbergChetan ParmarMartin ZenkeChia-Huei LinMartina ChiuChiara BrulloMartina RafanelliChiara GamberiMartina SperoChiara PanicucciMartínez CastelaoChiara PosarelliMarzena LaskowskaChiara StoccoMarzieh SalimiChiara VillaMasahiko TsuchiyaChien Chen ChouMasahiro KitamuraChien-Chang KaoMasahito FushimiChih-Hao LinMasahito NakanoChih-Peng LinMasanao NakamuraChih-Sung LiangMasanori MurakamiChin-Ping KungMasataka SumiyoshiChi-Ting HorngMasataka SunagawaChizu SanjobaMasatoshi NakamuraChris FinkemeierMasayoshi KuboChrissa SiokaMassimo BusinChristian A. MuellerMassimo CapocciaChristian KosanMassimo GeunaChristian MackeMassimo GrilliChristian Rønn HansenMassimo RuggeChristiane GerardMatei BratuChristina BinderMateja Dolenc VoljčChristine M. MermierMateusz JankowskiChristoph BurgerMateusz PuśleckiChristoph CentnerMathieu NacherChristoph ThomssenMati PääsukeChristopher F. TenggardjajaMatija BarbičChristopher J. YoungMatjaž JerebChristopher John LundstromMatteo BrucoliChristopher S. SnyderMatteo FerroChristos ChatzikyrkouMatteo FlorisChristos EftychiouMatteo GhilliChristos TsalikidisMatteo LucchiniChrysovalantis KorfitisMatteo Luigi Giuseppe LeoniChung Sim LimMatteo NardinChung-Feng LiuMatteo ScopettiChun-Hou WangMatthew G. K. BeneschCino BendinelliMatthew J.S. ParkerCiprian TomuleasaMatthew NeedlemanCiro SalzanoMatthew R. McCannClare Gordon-ThomsonMatthew ReedClaude FranceschiMatthew T. PatrickClaudia Hube-MaggMatthias BuechterClaudia IsonneMatthias GatzClaudio PuscedduMatthias KraemerClaudio ZuccaMatthias TroeltzschClaudiu MorgovanMatthias ZeilerCleland Heather J.Mátyás MeggyesClifton HolmesMaud A. W. HermansConcetta De PasqualeMaura M. ZyllaConcetta Di NoraMaura NicolosiCord HuchzermeyerMaurizio Cappelli BigazziCorneliu Petru PopescuMax Carlos Ramírez SotoCorrado BernasconiMaximiliano Tamae KakazuCorrado CampochiaroMaxwell SuCosimo Di RaimondoMay BisharatCostantino SchiaviMay Portuguez CastroCourtney A. MarshMayur ChoudharyCraig PearlMd Jamal UddinCristian FurauMeda-Lavinia NegrutiuCristian NavarreteMeerambika MishraCristián OyanadelMeghan B. BrennanCristian ScheauMehrdad EmamiCristiano Pedrozo VieiraMejía CarmenCristian-Radu JecanMelanie PlinsingaCristina Anna Maria Lo IaconoMelis PalamarCristina BarosaMelissa BaggieriCristina CarbonellMelvin R.Pete HaydenCristina ChircovMengxi ShenCristina López CadenasMercedes GinerCristina M. Fernández-DíazMia RakicCristina Martin-SabrosoMicah B. LeshemCristina MondelloMichael AntoniouCristina PopMichael ChristCristina SegoviaMichael D. StaehlerCristina SisuMichael FrassCristina SkrypnykMichael GreenCristina-Mihaela LăcătușuMichael Michael SamarkosCristos IfantidesMichael MielewczikCsiba LászlóMichael NekludovCuilan LiMichael PoteserCyrus MotamedMichael ScharfschwerdtDae Ho LeeMichael SchwakeDagmar PavluMichael StarkDaisuke InoueMichael T. KoltzDaisuke SatoMichael WallerDale Wilson ChapmanMichaela FrolíkováDalila ScaturroMichail DiakosavvasDamian HudziakMichał ArkuszewskiDamien VitielloMichał BiernackiDamien YoungMichał P. PlutaDana BadauMichał StasiowskiDana PopMichał ZimeckiDana StoianMichalina BłażkiewiczDanguole SatkunskieneMichel Kabamba NzajiDaniel A. BoullosaMichele ArcopintoDaniel B. DrachmanMichele BoffanoDaniel BléroMichele CampigottoDaniel JostMichele GaffuriDaniel OhMichele LorenzoDániel PécsiMichele PaciottiDaniel RaffertyMichiko ShimodaDaniel Robert SchlattererMichiyo YamanoDaniel ŚlęzakMiglė BacevičienėDaniel TurudićMiguel Angel Sanchez-TenaDaniel VanekMiguel Garcia JaenDaniela BoccoliniMiguel MontoroDaniela Maria MateiMihaela MoscaluDaniela PereiraMihai Dorin VartolomeiDaniela PiancatelliMihai Emil CăpîlnaDaniela ZuccarelloMihai JuncarDaniele ArmocidaMihai NitulescuDaniele CafollaMihai Stefan MuresanDaniele CastellaniMihail TodirasDaniele D’AlonzoMihnea-Alexandru GamanDaniele LinardiMikael LehtihetDaniele MasaroneMikel MikhailDaniele PironiMikhail DubininDanilo CialoniMiki KushimaDanko MiloševićMikyoung JunDanuta PentakMilagros Carrasco-PratsDaphna DrorMilena DeptułaDario BruzzeseMilena ŚwitońskaDario CalafioreMiloš MaksimovićDario TrapaniMin Young LeeDariusz KotlȩgaMina SamukawaDariusz ŁątkaMinas E. PaschopoulosDariusz WaniczekMinato NakazawaDavid A. EdelmanMing ChenDavid Alexander Christian MessererMingchao HuangDavid Alves FerreiraMing-Feng WuDavid BellarMiquel QuerDavid C. GazeMircea-Catalin FortofoiuDavid ChitayatMirco SchapherDavid F. MobleyMirella FalconiDavid GendelbergMirna ŽulecDavid GreenhalghMiro JukićDavid HerzogMiroslav HercegDavid J. HalsallMiroslava PřidalováDavid L. MayorMirto FolettoDávid LíškaMirza PojskicDavid Machado-ArandaMisa HiroseDavid ManninoMitsuru FutakuchiDavid ObertMi-yeon EunDavid OliverMohamed AliDavid Richard WoodsMohamed AlsharediDavid S. WeberMohamed ElessawyDavid SanterMohamed KhettabDavid SloukaMohammad Al-Mahdi Al-KaragholiDavid T. LiuMohammad Esmaeil BarbatiDavid VizcayaMohammad Jamshed SiddiquiDavide BavaroMohammad KamranDavide BizzocaMohammad NusairDavide BorroniMohammed A. Al-AbriDavide DonnerMohammed Nader ShalabyDavide LugliettoMohan RudrappaDavide RaineriMohit PahujaDawid StormanMoises Leon-JuarezDebbie Joffe EllisMojca BizjakDebora RosaMoktar ChtaraDeborah J. BowenMonali SwainDeepak OzhathilMonia Di PreteDeiana RomanMonica D. PrakashDejan GeorgievMonica TerenzianiDejan ReljicMonika KoziełDèlia ReinaMonika Łopuszańska-DawidDemetra SocolovMonika SitarzDemetrio LarraínMonika UlamecDenis MalaiseMorales Cano DanielDenisa MarginaMoshe Rav AchaDenisa VojtassakovaMosiewicz JerzyDenise Paschoal SoaresMotohiro MunakataDennis NiebelMuhammad E. HaqueDennis ThomasMuriel MathonnetDeodato AssanelliMustafa JalalDerek CostelloMustafa Zelal MuallemDeshayes SamuelMyung-Gwan KimDevrim Pesen-OkvurNabil EbraheimDhananjay YadavNadia NajafiDhanush HaspulaNadja FreundDharam AblashiNaga SamjiDharani HapangamaNageswara PilliDhaval ChauhanNahum RosenbergDhiraj P. MuraleNaiara OzamizDhiran VergheseNai-Jen ChangDiana HeimesNajmeh (Nancy) JafariDiana MartinsNakano YusukeDiana RamasauskaiteNana Kwadwo BiritwumDiana ŽaliaduonytėNancy LonsdorfDiego IaconoNaohiko ImaiDiego LopsNaoki AsanoDiego ManavellaNaoki HashizumeDiego RaimondoNaoki SatoDimitri PoddigheNaomi J. Serling-BoydDimitrios K. FilippiadisNaomi NakayamaDimitrios KaragiannakisNapoleão Martins Argôlo NetoDimitrios PanagopoulosNardelli CarmelaDimitrios PatouliasNarendar DudhipalaDimitris LambrouNat LenzoDiogo MonteiroNatalia BelosludtsevaDishary BanerjeeNatalia DłużniewskaDivya GopinathNatalia DowgiałłoDmitrii A. RogatkinNatalia Maria SerwinDmitriy V. MazurovNatalia PawlasDmitry KhochenkovNatalia Saldaña-GarcíaDo Gyun KimNatalia SzejkoDo Hoon KooNatalie EnninghorstDoga KuruogluNatasa Bogavac-StanojevicDoina AzoicaiNataša Marčun VardaDomenico AzzolinoNathalia Fernandes FagundesDomenico DalessandriNathalie MorelDomenico LeporeNathanael James YatesDominik KraljNavin Kumar DevarajDominik PförringerNebojša LalićDomiziano TarantinoNenad PandakDong Hoon LeeNenad VukojevićDong Hui LimNibedita PatelDong Wook ShinNicholas D. KlemenDong-Hee RyuNicholas F. FitzDong-Hyun KimNicholas J. AndersenDongmei LiNicholas ObermüllerDongWoon HanNicola FuscoDora SedmakNicola MarottaDorota Iwaszkiewicz-GrzesNicola MartucciDorota Olex-ZarychtaNicola MontemurroDorota RaczkiewiczNicola TartagliaDorret I. BoomsmaNicola ValèDr. Nishantha NanayakkaraNicolae GicaDragan KovačićNicolas PaulDrago JelovacNicolas Perez-FernandezDragos Catalin JianuNicole Isolde Zur NiedenDragos SerbanNicole N. HaeseDrahomíra SpringerNicoletti GiovanniDubravko HabekNidal JaradatDulce EstêvãoNidhi KapoorDušica L. MarićNihal ÇetinDušica PahorNikitas NikitasE Scott SillsNikola StefanovićEbrahim Abbasi OshaghiNikolaos MachairasEbtesam Al-SuhaimiNikolaos SiafakasEdgar SchäferNikolay Margaritov RunevEdison JahajNikos GorgoraptisEdith ClarosNina RembiałkowskaEdmundo Rosales-MayorNiranjan KhadkaEdmunds ReineksNisarat RuangsawasdiEdoardo BraunerNitish GulveEdoardo FranciniNoah MoruzziEdouard GerbaudNobuhiro TanabeEduardo CelentanoNobuto NakanishiEduardo Flores-UmanzorNorbert GrafEduardo Molina-JijonNorbert NighoghossianEduardo OliverNorbert SchrageEdurne Úbeda DocasarNorbert StefanEdward Andrew TownsendNoreen GleesonEdward J. PavlikNorihiko KikuchiEdyta WlaźlakNorio YamamotoEdyta ZbrochNorma CameliEfstathios D. PagoureliasNorma G. Padilla-EguiluzEglė KontrimavičiūtėNoura DosokyEhsan NazemalhosseiniNoureddin Nakhostin AnsariEiichi AkiyamaNovak TiborEiichi WatanabeNunzio VelottiEirini GrapsaO Kyu NohEitaro KodaniOana Gabriela DimienescuEkaterina IvanovaOctav Marius RussuEleanor J. CheadleOctavian EnciuElena M. MarrónOfra WalterElena Martinez-SanzOgnjen BarcotElena MontiOhsawa SuguruElena PeevaOhsuke MigitaElena RossiOk Hyung NamElena-Mihaela CordeanuOlaf EberhardtEleni KotsiomitiOlatz ZenarruzabeitiaEleni PetkariOlga KruszelnickaElettra MerolaOlga MatosEleuterio A. Sánchez-RomeroOlga OstrovskyEliana CostanzoOlivier BergesEliana LacerdaOlivier BousigesElion HoxhaOlivier GarbinElisa BelluzziOlivier LairezElisa CairrãoOlle StendahlElisa RossiOmar KujanElisabetta CocconcelliOmer TrivizkiElisabetta RazzuoliOndrej OndičEliza WasilewskaOreste GalloElizabeta B. Mukaetova-LadinskaOriol Cantó-NavésElizabeth DraganovaOsagiede OsayandeElizabeth M. BroadOscar Figueras-AlvarezElizabeth R. LusczekOskars KalejsEllen FunkhouserOsman AktaşEllen HillegassOsman ÇekıçElton DajtiOsman UlviÉlvio Rúbio GouveiaOyuna KozhevnikovaElżbieta CiporaPablo Diez-VillanuevaElżbieta Łodyga-ChruścińskaPakcheong ChowEmanuel SchwarzPalaniappan RamanathanEmanuel VamanuPaloma Barjola ValeroEmanuela FinaPanagiotis AthanassiouEmily K. TarletonPanagiotis XaplanterisEmmanouil V. DermitzakisPandurang KolekarEmmanuel NicolasPankaj PandeyEmmanuel RineauPaola GandiniEnisa ShevrojaPaola MartinganoEnric ReverterPaola QuaresimaEnrico BroccoPaola SimeoneEnrico GiordanPaolo BoffanoEnrico Mario CamporesiPaolo BrusiniEnrico PolaPaolo CalabròEnrique Rodríguez-BorjaPaolo DolzaniEnrique SantasPaolo LimoliEri KuboPaolo Matteo AngelettiEric De GrootParmenion TsitsopoulosEric HermandPascal BauerEric Lodewijk StaalsPascal Le CorreEric YoshidaPasquale CianciErica MenegattiPasquale Di MaioErik BehringerPasquale LongobardiErik H. F. M. Van Der HeijdenPasquale PiconeErtan SaridoganPasqualino SirignanoErwan MortierPasupathi SundaramoorthyErwin De BruinPat R. VehrsEstefania Lozano-VelascoPatrice ForgetEstefania NuñezPatricia Correa-GhisaysEsther A. BaloghPatrick G. BradyEsther Monika SpeerPatrizia LoPrestiEszter FüzékiPatrizio MazzoneEtsuko MiyagiPaul A. ImbachEugene KimPaul Friedrich EngelhardtEugene YuriditskyPaula BoaventuraEugenio ProvenzanoPaul-Mihai BoarescuEun Kyoung LeePaulo Magno Martins DouradoEungseok OhPaulo PalmaEunice CarrilhoPaulo Ricardo De SouzaEva BagyinszkyPavan UpadhyayulaEva ČeškováPavel CaldaEva KoetsierPaweł KrzyżekEvaldo FaviPaweł WańkowiczEvelyn BlumenbergPaweł ZalewskiEverett F. MagannPayal M. ShahEvert F.S. Van VelsenPayam SadeghiEvgeni BrotfainPedro Gil-MadronaEvgenia CherouveimPedro MorouçoEwa SobolewskaPedro Vieira BaptistaEwa TomaszewskaPetar ĐanićFabian SchwendingerPetca Razvan-CosminFabiana LucàPeter CnuddeFabio CacciapagliaPeter H. SchurFabio FabbianPeter J. BeekFabiola PaiarPeter JonesFabrizio DamianoPeter MesenbrinkFadi I. MusfeePeter ParryFady ShararaPeter ProdingerFahd AlhamdanPéter TörökFahimeh Alsadat ShojaeiPetr O. IlyinskiiFaisal F. HakeemPetr StastnyFakhraddin NaghibalhossainiPhil J. HandcockFangshi ZhuPhilip W. WertzFaramarz Ismail‐BeigiPhilippe ColleryFaris F. BrkicPhilippe MarchettiFaris G. ArajPhilippe Matthias TschollFayez AlshamsiPhilippe Von RothFeby SaviraPhotios A. AnninosFederica Di SpiritoPia Clara PafundiFederica FurfaroPier Camillo ParodiFederica IlardiPierachille SantusFederica Lo SardoPiergiorgio DanelliFederica LovisaPiergiorgio IannoneFederica PezzutoPierre LafèreFederica PianiPieter A. BothmaFederica RossoPil-sung YangFederica VeronesePingal DesaiFederico FerrariPing-Fing ChiuFedor LuriePiotr CzarneckiFelice BedfordPiotr Henryk SkarzynskiFelicia Fei-Lei ChungPiotr KalicińskiFelipe ÁvilaPiotr LadyzynskiFelix AbererPiotr MorasiewiczFelix Campos-JuanateyPiotr PoznanskiFelix ClanchyPiotr RolaFelix GoeserPiotr WaleckiFélix Marcos TejedorPiotr WychowańskiFelix RommelPitoyo HartonoFelix W. A. WaibelPiyush BaindaraFerdinando AgrestaPojen KoFerdinando Carlo SassoPonnusamy SubramaniamFernández-Codina AndreuPooi Ling MokFernando BrilPourya GholizadehFernando Capela E SilvaPrabhu MathiyalaganFernando GilsanzPrachi GuptaFernando GõmezPradeesh SivapalanFernando HernanzPrakash PeddiFernando LõpezPranshu SahgalFernando Martinez TaboadaPrasanna P. MithraFernando SantosPrerna VarmaFevzi Fırat YalnızPriscila GiavedoniFilipe Manuel ClementePriyanka VyasFilipe PereiraPunam Prasad BhadaniFilippe Falcao-TebasPuvanalingam AyyaduraiFilippo TorielloPuy GarrastachuFirdaus HayatiQi JinFlor H. PujolQian ZhangFlorian GesslerQin FuFlorian KraftQu DengFlorin OnisorR. KocyłowskiFortunato IacovelliR. M. HermannFotios DrakopanagiotakisRabia Sannam KhanFotis TsetsosRade M. BabićFouquet GuillemetteRadosław KempińskiFrances Meier-GibbonsRadu-Stefan RomosanFrancesca AmorosoRafał MorgaFrancesca M. DimouRafal MoszynskiFrancesca MorettiRafal NowakFrancesca RizzelloRafał PęksaFrancesco AddevicoRafał PokrowieckiFrancesco BennardoRafał WatrowskiFrancesco BiancoRaffaele GalieroFrancesco ChianconeRaffaele GiordanoFrancesco CoralloRaffaele OrnelloFrancesco CorattiRaffaele SerraFrancesco FeoRafiou AgoroFrancesco FrattiniRaja Gopal Reddy MooliFrancesco MeliRajamma MathewFrancesco Saverio SorrentinoRajiv DahiyaFrancesco SignorelliRajiv DoddamaniFrancesco VerdeRajkumar Kottayasamy SeenivasagamFrancine DurocherRajnish SahuFrancis DumlerRakhi IssraniFrancisco Espinoza-GomezRalf ErberFrancisco Javier Rodríguez-LozanoRalf RegenthalFrancisco José Rodríguez VelascoRalph FingerhutFrancisco Rodríguez-HurtadoRalph GeorgeFrancisco Vera-SempereRaluca DumacheFranco FulcinitiRaluca Iulia JuncarFrancois Alhenc-GélasRam Kumar SelvarajuFrancois CornelisRam SharmaFrancois EyskensRamesh PandeyFrançois RoubilleRami S. KantarFrank ComhaireRamin SalimnegadFrank DresslerRamon SirventFrank PaulsenRamona PalomboFrank SanderRamy Abou GhaydaFrank SuhrRan HarelFranz RoedelRan LadorFrede DonskovRana K. ZabadFrieder C. KrafftRandal PhamFulvio BorellaRania HathoutFumitaka KogaRanjeev Chrysanth PulleGabriel Claudiu TenderRanjith Menon MuraleedharanGabriel Dominguez-MaldonadoRanko ŠkrbićGabriel Gijón NoguerónRaquel CostaGabriel StefanRasa SabaliauskaitėGabriela AniteiRasmus Bo JansenGabriele CervinoRasmus RiviniusGabriele Di GesaroRathi Pravin MotilalGaetano AlfanoRatko PrstačićGaetano GalloRaúl Reina VaílloGaetano IsolaRavi Philip RajkumarGaia PellegriniRavin RatanGaia SampognaRay MannehGardar ArnasonRayane Brinck TeixeiraGary R. SmerdonRazvan SocolovGary S. LeiserowitzRebecca E. HoedemaGaston KapukuRebecca M. SchurGaurav GhagRebecca PrevotsGavriel ChaushuRebecca RoedigerGégout-Petit AnneRecai ErgunGema Martinez-NavarreteReda ŽemaitienėGennaro MartucciRedi RahmaniGeorg BöningReima SuomiGeorg StüssiReinhard KoppGeorge AntonogeorgosRenata GrzywaGeorge BazoukisRenata Voltolini VelhoGeorge C. BroukhanskiRenata ZajączkowskaGeorge Calin DindeleganRenato GondarGeorge D. ChlorosRené HartensuerGeorge D. VavougiosRené R.H. HartensuerGeorge LatsiosRene Van EsGeorge P. ParaskevasRene VerdonkGeorgi PopivanovRen-Jay SheiGeorgia KarpathiouResi PucciGeorgios D. PanosReto BabstGeorgios ManikisRhea BhargavaGeorgios TziatziosRian Q. Landers-RamosGerado Alvarez-UriaRičardas RadišauskasGerald H. TomkinRicardo J. JoseGerard AnmellaRiccardo DraghiGerd Marie AleniusRiccardo FerraciniGerd U. AuffarthRiccardo MastroianniGerhard JungRiccardo Matteo CioniGermain HonvoRiccardo SarzaniGerwin Alexander BernhardtRiccardo VioGhada Hussein NaguibRichard D. FeinmanGheorghe Nicusor PopRichard GunGheorghe PeltecuRichard KdolskyGiacomina BrunettiRichard KonesGiacomo BriscaRichard KovacsGiacomo FarìRichard WellerGiampaolo MontesiRick KampsGiampiero ParrinelloRimantas VilcinisGian Andrea BindaRita PavasiniGian Franco ZannoniRittu HingoraniGian Luca SfasciottiRoald Flesland HavreGianCarlo PecorariRobert A. ConvissarGiancarlo RipabelliRobert B. PernaGianfranco CervellinRobert C. DempseyGianluca LuccheseRobert Cristian PurdoiuGianluca M. TartagliaRobert CzabanskiGianluca RizzoRóbert HalmosiGilles LesieurRobert HoerrGillian M. BarrattRobert J. BrownGillian SolesRobert K. ClemensGiordano AlessandraRobert Krawczyński MaciejGiorgi KuchukhidzeRobert NaeijeGiorgio BoganiRobert SabiniewiczGiorgio LofreseRobert SitarzGiorgio MatteiRoberta FenoglioGiovanna FerraraRoberta GasparroGiovanna FranciosaRoberta ImperatoreGiovanni BocciaRoberto CalistiGiovanni ChiariniRoberto LatiniGiovanni CillinoRoberto Lo GiudiceGiovanni CobellisRoberto N. MirandaGiovanni DamianiRoberto S. FrancoGiovanni GaleotoRocio Alejandra Chavez-SantoscoyGiovanni IolasconRocio Garcia-RubioGiovanni MatareseRodrigo Fernando PesántezGiovanni MisseriRodrigo Saar GomesGiovanni PaolinoRoelf S. BreederveldGiovanni PapaRoger P. HarrieGiovanni TarantinoRoger RochatGiovanni ZoccaliRohita SinhaGisella GuidoRomain BourcierGiulia BivonaRoman LeischikGiulia BriganteRoman NowickiGiulia SciosciaRoman S. NagovitsynGiuliana ScarpatiRomeo Petru DobrinGiulio Di MizioRonen PerezGiulio Francesco RomitiRonghua ZhuGeGiuseppe AmorusoRoopma WadhwaGiuseppe BorianiRosa M. MartiGiuseppe CaminitiRosa Pérez-BadiaGiuseppe D’AngeloRosaura Esteve-PuigGiuseppe Di PietroRoss P. HolmesGiuseppe ForteRossella BrunoGiuseppe GiustiRoustem MiftahofGiuseppe MaccagnanoRuben Yap KannanGiuseppe MagliuloRudolf KuklaGiuseppe MarongiuRuediger E. ScharfGiuseppe MasciaRui HenriquesGiuseppe RivaRuna LindblomGiuseppe SchepisiRupendra ShresthaGiuseppe StabileRupesh KumarGiuseppina CampisiRuxandra Diana SinescuGloria CalagnaRyan KennyGo KawanoRyo MaekawaGokhan OzunerRyoko InakiGonzalo J. RevueltaRyong Nam KimGoohyeon HongRyuta TakenakaGoran CuricS. Paul OhGöran LindSa Ra LeeGordana PavlišaSabina LimGoro ShibukawaSabine LinsenGoutam MondalSabine ZittaGraham PluckSabrina AnticoliGrant R. KolarSachit AnandGraziella BonettiSaeid Abbasi-MalekiGrażyna JerzemowskaSafak Guel-KleinGrażyna SygitowiczSagrario Pérez-De La CruzGregor ProsenSajjad ShiraziGry Kjaersdam TelléusSalamah AlwahshGrzegorz R. JuszczakSaleh S. BaeesaGuglielmo ManticaSalvador PérezGuido VielSalvatore BocchieriGuillaume E. CourtoySamara RiceGuillaume GiraudSamet ÖzdemirGuillaume LefebvreSamhati MondalGuillermo LaheraSami M. Abd ElwahabGuillermo PlazaSami ViskinGuinevere GraniteSamina AlamGulbin Sahender AygencelSamson N. DowlandGulzhanat AimagambetovaSamuel Fernández CarneroGunnar B. KristensenSamuel M. SilvestreGünter EmonsSamy M. RiadGunter LauxSandeep ThannaGurdeep MarwarhaSandra KalthoffGurpreet Surinder GandhokeSandra Maria BarbalhoGyeong Eun MinSandra MilićGytė DamulevičienėSandra Santos-MartinezH. Denis AlexanderSandra Sumalla-CanoH. Ray GambleSang Ki LeeHacer Dogan VaranSang-gyung KimHaewon ByeonSangu MuthurajuHagit SalemSanja DespotovićHailun ZhanSanja HromišHaipeng LiuSanja Popović-GrleHaishu LinSanja Zoričić CvekHåkan AlfredsonSanjaya Kumar SahuHalina JurkowskaSanjeet Singh Avtaar SinghHan Naung TunSanoj RejinoldHanaa SallamSantiago BallazHanatsu NaganoSantosh PandaHank WhiteSara BozziniHans ChristiansenSara LiguoriHan-Sin JeongSara RattottiHans-Peter SimmenSara RicciardiHarald H. KesslerSarah ThomisHariharan RajuSarah WatsonHarneet AroraSarina K. MuellerHarold L. RekateSarka JelinkovaHaroon MajeedSarumathy SundararajanHassan DastsoozSascha K. ManierHassan KassassirSaska RoskarHattanas KumchaiSatadru LahiriHaytham A. SheerahSatoko OsukaHeather TickSatoko YamawakiHefei ZhaoSatoshi IgawaHeidi SowterSatoshi MiyamuraHeiko RuehlSatoshi YamaguchiHelena BarrasaSaulius SvagzdysHelena Fernández LópezSavalan Babapoor-FarrokhranHelena MartynowiczSaverio LatteriHelene VossosSchiavone MarcoHelle Smidt MogensenScott OkunoHelmut MairSean G. McKennaHemant K. MishraSebastian BjörklundHemant KulkarniSebastian GnatHeming YaoSebastian J. FlackeHendrik ReynaertSebastian JohnstonHenk KoppelaarSebastian O. DeckerHenrik C. BäckerSebastian SchadeHenrik FalhammarSebastian ScheidtHenrique NascimentoSebastian WawrockiHenry SeligmanSebastien RubinHenry SutantoSebastjan BevcHenryk DomanskiSedighe KarimzadehHerman T. DepypereSeisuke OkazawaHervé ReychlerSelami Aykut TemizHesam HashemianSergio CarandinaHiba NatshehSergio CoccheriHidemi NakataSergio De SalvatoreHideo SuzukiSergio SalernoHidetada FukushimaSergio Zaccaria ScalinciHieyong JeongSerkan VarolHiginio González-GarcíaSeth M. TarrantHimanshu AroraSeth TarrantHinne A. RakhorstSeyed Behzad JazayeriHiran A. PragSeyedeh-Sara HashemiHirayuki EnomotoShagun KrishnaHiroaki MurataShahram MolavynejadHiroaki TanakaShamik ShahHiroki KitakataShankar Jaikishan EvaniHironaga SatakeSharmila FagooneeHiroshi FukushimaShashank SrivastavaHiroshi WakuiShashikant SholapurkarHiroshi YasudaShaun J. KiltyHirotaka MiyamotoShaun SabicoHirotaka TanabeShaw WatanabeHiroyoshi MoriShigeaki SuzukiHiroyuki WakiguchiShigekazu SuginoHisao ImaiShigeki MatsubaraHitesh VijShigeki YamaguchHitoshi KobataShigeo IijimaHitoshi MaemotoShigeru AokiHitoshi MomoseShin-Cheh ChenHoang HaiShing-Hwa LiuHonghe LiuShingo KuwataHongwei HanShingo YamamotoHongyan ChaiShin-hoo ParkHorace MassaShinya MatsuzakiHoward SelingerShin-Yi ChiouHrvoje BudinčevićShiqiu MengHsin-Yao WangShitiz SriwastavaHu ChenShreesh SammiHuafeng WangShreya TocaciuHubert Van Den BerghShu-hao HsuHui-Ju LinShuji OzakiHui-Ting YangShungo EndoHumberto HernandezSiddharth DugarHungwen ChenSiddharth ThakerHun-Gyu HwangSiegmund LangHunter BennettSigita BurokienėHyeong Won YuSigita GlaveckaitėHyuck Jin LeeSigrid Müller-DeubertHyung Joo SuhSihong SongI. Medina PorqueresSik NamgoongIban AldecoaSilvia AziriovaIbrahim M. SayedSilvia BianchiIbtihal Al AmriSilvia D’AgostinoIgnacio SegarraSilvia De Miguel-BilbaoIgnacio Torres-NavarroSilvia M. Sanz-GonzálezIgor DumicSilvia RiondinoIgor LocatelliSilvia RocchiccioliIkchan JeonSilvio BorrelliIl Seok ParkSimon N. RogersIlaria AttiliSimon PodnarIlaria Tocco TussardiSimona BungauIlario Giovanni RapposelliSimona CensiIlavenil SoundharrajanSimona GaberščekImena RexhepiSimona NicoaraInaki ElioSimone MorraIñaki PermanyerSimone MorselliIndrikis KramsSinagra EmanueleIndu G. RajapakshaSira NanthapisalInés Aguinaga-OntosoSkand ShekharInês Morais CaldasSlavoljub ŽivkovićInge A. H. Van Den BerkSlobodan V. MićovićInnocence HarveySmaragdi MarinakiIn-Ryoung KimSmeeta SardesaiIoan LascarSneha GautamIoan SarbuSnežana Tomašević TodorovićIoan Stefan FlorianSnježana Mardešić-BrakusIoannis FlessasSonali NashineIoannis KyrgiosSongkwan SilaruksIoannis LiampasSong-Lih HuangIoannis Ntanasis-StathopoulosSonia D’ArrigoIoannis VamvakarisSönke HarderIoannis VourosSoo-Min OkIonut DonoiuSorin HostiucIonut LuchianSotirios ApostolakisIosif MarincuSoumojit PalIrena Duś-IlnickaSoung Min KimIrena TušerSousa David CordeiroIrene Cozar-CastellanoSousana PapadopoulouIrene González-MartíŠpela BogatajIrene Pina-VazŠpela KonjarIrene SchiavettiSpomenka ManojlovićIrfan A. RatherSpyridon ChytirisIrina RistescuSrđan NovakIris UdasinSrdjan B. AleksandrićIrma Magaly Rivas SernaSridhar MuthusamiIrteja IslamSrinivas PittalaIrwin KleinSriram SundaravelIrzal HadžibegovićStacy Dawn ThompsonIsabel Escobio-PrietoStamatis GregoriouIsabel LegazStamatis KarakonstantisIsabelle Ruchonnet-MétraillerStanescu Ana Maria AlexandraIsam BsisuStanislav M. CherepanovIsao OtsukaStanislav PekarskiyIssam TanoubiStefan Cristian VesaIstván OsztheimerStefan HarsanyiItalo AngelilloStefan J. LangIulia-Cristina BagiuStefan M. FroschauerIvan ĆukStefan MihaicutaIvan StojanovićStefan RoosIvana BajkinStefano BallestriIvana DjuricicStefano CanosaIvana HálováStefano Da PozzoIvana KholováStefano Francesco CrinòIveta MintāleStefano GuadagniIvica GrgurevicStefano GuminaIwona Olszewska-CzyżStefano Marco Paolo RossiIwona Poziomkowska-G ˛esickaStefano Maria PriolaIzabela Mauricio RezendeStefano MariniIzabela ZakrockaStefano SelvaggioIzabella Karska‐BastaSten HellströmJ Alexander De RuStepan PodzimekJacek BucznyStephan LindemannJacek SzymańskiStephan PayrJacob BarStephan Van EedenJacob GeniziStephane RetyJacobo Rodríguez-SanzStephen David MyersJacobus PrellerStephen InnsJacopo BurrelloStephen LuzioJacqueline M. BentelStephen OpatJacqueline W. MaysSteve CarlanJadwiga HamułkaSteven P. EngebretsonJae Hoon MoonSteven Paul NisticòJae Sung LeeSteven T. PeperJae-Ho LeeStuart F. QuanJaehoon ChoSuchitra JoshiJaewoo KwonSudhir KotnalaJae-Young LeeSue GordonJai Young ChoSue R. PavordJaime Calvo-AlénSuhail AkhtarJain PriteshSukumaran AnilJakov MihanovicSulaiman S. IbrahimJakov MilićSun HuhJakub MorzeSundeep SinghJames HebertSung Eun KimJames HongSung-Hyoun ChoJames OschmanSung-kyu KimJames V. AquavellaSungmin LimJames W. SonneSusan ClarkeJames WilliamsSusana Del Pozo De La CalleJamie M. BogleSusana García-SilvaJamir Pitton RissardoSusana MonereoJan BilskiSusanne Dam NielsenJan BroggerSuzanne FredericksJan Jaap H.M. ErwichSyed Arman RabbaniJan Jacek KaczorTabito KinoJan KonarskiTae Han KimJan MieszkowskiTakashi HashimotoJan UlfbergTakashi MatsusakiJanaki DeepakTakashi OginoJanani IyerTakashi OhtsukaJane Ru ChoiTakehiko MatsushitaJane YardleyTakenobu YamamotoJanusz GodlewskiTakeshi KannoJaques WaisbergTamai KojiJarmila CelakovskaTamara Alempijevic MilovanovicJarosław BilińskiTanja J. MiličićJarosław KobielaTatsuya ArakakiJarosław PasekThierno Madjou BahJarosław PinkasThomas CluzeauJasbir MakkerThomas PolakJasbir UpadhyayaThorsten GuehringJasmine SinhaTillmann WetterlingJason C. SluzevichTiziano TestoriJason Kihyuk LeeTobias PiegelerJasper VergutsTom BschorJaspreet BhanguTomas KollerJavier CorralTomasz RakowskiJavier Merino-AndresTommaso ErcoliJavier Molina-CerrilloTomoaki MiyagawaJay S. MishraTomofumi NishinoJayarama GunajeTomohiro ChibaJayson StoffmanTomohiro MizunoJean Baptiste PialatTomokazu ShimizuJean GoldingTomomitsu MiyagakiJean VanderpasTomoyuki FujiokaJean-Louis PrudhonToru HifumiJean-Loup BascandsToshihiro NishizawaJeanne BertolliTrifan Anca-VictoritaJean-Philippe BerteauTsai Chen LiangJean-Sébastien RougierUi-Lyong LeeJeffrey A. NiezgodaVijay AvinJeffrey D. LambertViktor ReshetnikovJeffrey D. WhiteVinayak M. JoshiJeffrey M. WilsonVincenzo ScuderiJeffrey SilberzweigVito CelaJelena MandićVladimir I. GridnevJelena Osmanović BarilarWalter J. FlapperJennifer L. SchieferWei-Chang HuangJenq-Shyong ChanWen-Hsuan HouJens Christian MöllerWen-Wei SungJeong-Hoon LimWesley KephartJeroen Van SchependomXiangpeng LiaoJérôme LaurinXuqiao ChenJerzy B. GajewskiYajuan SuJerzy ChudekYang Jae WookJerzy WalochaYasunori OtowaJessica M. TurnerYasuyuki NagasawaJesús DevesaYauheni ZhalniarovichJesús Mena-ÁlvarezYawei LiJesús Molina-MulaYehuda ShienfeldJesús Oliva-Pascual-VacaYen-Chien LeeJesús PastorYin-Chih FuJhen-Wei RuanYoichi SakuradaJia-Feng ChangYoji NagashimaJiajia SongYongseok JeeJianyou GuoYong-Soo BaekJiffin K. PauloseYoshihiro FukumotoJingyi ZhaoYoshihiro IkuraJinhwa JungYoshi-Ichiro KamijoJin-kyun ParkYoun Joung ChoJinlu YuYoung-Joon JunJinse ParkYoungsook BaeJin-Seok LeeYu-Ching WenJinyoung ByunYuichi SaitoJiří EhrmannYuichiro NoiriJiro KishimotoYuji KabasawaJiro TeradaYukio MaruyamaJitendra KanaujiyaYuksel AltinelJitendra SinghYung-Shun JuanJizhong ChengZehuan LiaoJo KatoZhipeng LiuJoachim Kirsch



